# Translational bioinformatics has now come of age: TBC 2012 collection

**DOI:** 10.1186/1755-8794-6-S2-I1

**Published:** 2013-05-07

**Authors:** Ju Han Kim

**Affiliations:** 1 Seoul National University Biomedical Informatics (SNUBI), Systems Biomedical Informatics National Core Research Center, Division of Biomedical Informatics, Seoul National University College of Medicine, Seoul 110799, Korea

## Introduction

Translational bioinformatics is a rapidly emerging field of biomedical data sciences and informatics technologies that efficiently translate basic molecular, genetic, cellular, and clinical data into clinical products or health implications. Translational bioinformatics is now even more powerful than ever. The revolutionary progress in both bioinformatics and high-throughput genomics based on the flood of nucleotide sequences and microarray data will eventually transform the current practice of medicine forever, including diagnostics, therapeutics, and prognostics.

Translational research is a paradigm for an alternative research approach to the dichotomy of basic and applied research. Its cross-disciplinary nature is a serious challenge caused by the compartmentalization of the current research training paradigm. Moreover, bioinformatics itself is interdisciplinary. To understand and correctly address the clinical needs for ultimately improving human health requires strategically-minded scientists, multi-skilled teams, and knowledge-driven data infrastructure for integrative analytics. Despite the barriers, linking the molecular world to the clinical world and vice versa will enormously benefit human health.

Since its inception, the Translational Bioinformatics Conference (TBC) aims to highlight the multi-disciplinary nature of the research field and provides an opportunity to bring together and exchange ideas between international translational bioinformatics researchers. The Second Annual TBC 2012 in Jeju Island of South Korea provided the opportunity to substantially improve the understanding of complex and rare diseases and proposed new ways of approaching basic health problems by integrating genomic and clinical data.

## Personalized genomic and physiologic signatures

Translational bioinformatics together with the '-omics' (genomics, transcriptomics, proteomics, and metabolomics) investigates the contribution of genes, transcripts, proteins, and metabolic pathways to human physiology and the variations of its sub-systems that can lead to disease susceptibility. While a plethora of genomic signatures have successfully demonstrated their predictive power with diagnostic and prognostic values in many cancers, these genomic signatures are, as Chen et al. (University of Chicago, USA) correctly pointed out, merely statistically significant differences between dichotomized phenotypes that are in fact severely heterogeneous [1].

Personalized medicine attempts to determine individual solutions based on the susceptibility profiles of each individual. Based on their previous research on FAIME (Functional Analysis of Individual Microarray Expression, [2]) method, Chen et al. generated pathway signatures for an individual patient and computed aggregate 'molecular-pathway' scores using rank-weighted gene expression values of an individual sample. FAIME not only improved the predictive power but also provided personalized pathway profiles that have potential for the personalized therapeutics of cancer.

To overcome the limitations of population-based medicine, both personalized physiologic and genomic signature are introduced in this issue. Grossman et al. (Stanford University, USA) constructed Spearman correlation networks of pairs of 29 physiologic variables measured at one-minute intervals from nineteen intensive care unit patients [3]. According to the connection changes in the presence and absence of pressor drug administrations, the networks can be divided into 'static', 'either-or', and 'sign-change' networks that permit individual patient-level analysis of physiological states. While the 'static' networks represent known normative physiological relations not associated with pressors, the others represent new physiological relations sensitive to pressors and/or to the reasons for giving the drugs.

## Dynamic network biomarkers and pathophysiological correlates

Biomarkers are indicators of biological states that are useful for the evaluation of physiological states, pathogenic processes, and therapeutic responses. Perturbations between two biological states, however, lie at the network level and not at any one molecular biomarker. Genomic data has the potential to reveal dynamic relations between genes during temporal progressions. While traditional biomarkers are mainly used to examine only the current disease status based on a single molecule, Liu et al. (Shanghai Institutes for Biological Sciences, China) defined a new type of biomarker, i.e., dynamical network biomarker, for marking the time period just before the catastrophic state transitions between normal state, pre-disease state, and disease state [4]. By using temporal gene expression profiles of non-obese diabetic mouse, Liu et al. successfully identified dynamical network biomarkers that distinguish the pre-disease state from the normal state and predict the upcoming disease onset of type-I diabetes by the theory of early-warning signals of complex diseases [5].

Penrod and Moore (Dartmouth College, USA) created temporal gene coexpression networks from sequential biopsy samples of breast cancer patients at diagnosis (pre-treatment), following 10-14 days (mid-treatment), and following 90 days of letrozole treatment (post-treatment) [6]. They showed that the breast tumor coexpression networks are extensively rewired to adapt to the perturbation in the course of letrozole treatment and identified biomarkers maintaining network integrity and controlling information flow. It is suggested that many of the key genes for modulating the information flow at each of three time points are specific to the perturbation conditions.

Network biomarkers can be directly associated with clinically significant phenotypes. Yim et al. (Ajou University, Korea) investigated the gene expression profiles of 23 patients with congenital muscular torticollis and 5 control tissues [7]. Differentially expressed genes and protein network modules were identified as the prominent biomarkers showing strong correlation with the disease severity quantitatively measured by pre-operational MRI images. Pathophysiologic mechanisms suggested by molecular genomic features can be well translated to gross anatomical features.

## Knowledge assembly for drug repositioning and rare variant interpretation

Public databases and numerous biomedical knowledge resources are invaluable for a high profile translational bioinformatics research. They lead researchers to a better understanding and interpretation of clinical and genomic data that cannot be achieved by numeric data analysis alone. Hard problems like drug repositioning and rare variant interpretation can be successfully addressed by systematically borrowing the power of biomedical knowledge assembly. Cheung et al. (University of British Columbia, Canada) predicted drug-disease associations by evaluating the over-representation of Medical Subject Heading (MeSH) terms for chemical compounds assigned to diseases- or symptom-related research publications in the MEDLINE database [8].

Moore et al. (Vanderbilt University, USA) developed a flexible collapsing method guided by biological knowledge and created a comprehensive biomedical knowledge repository (LOKI, Library of Knowledge Integration) for supporting systematic multi-level binning of low frequency DNA variants for association testing [9]. LOKI determines the boundaries of the bins for rare variants by functional regions, evolutionarily conserved regions, genes, pathways, and/or intergenic regions in a flexible and user-defined fashion. One can choose statistical tests according to the hypothesis being tested for the understanding of the role of rare variation in complex human disease.

## Methods for further translation

Better bioinformatics tools and methods are required for a successful translational research. In this issue, advanced solutions for well-known bioinformatics problems were introduced. Kim et al. (CHA University, Korea) proposed a novel algorithm to combine haplotype clusters for diplotype-based association studies [10]. Van de Wiel et al. (VU University, Netherlands) developed a Baysian method for shrinking multiple parameters in a statistical model [11]. They applied the method to high-throughput RNA interference screening experiments to overcome the small sample size by borrowing information across the feature dimension. Kim et al. (Sookmyung Women's University, Korea) extended the multifactor dimensionality reduction (MDR) algorithm that is currently limited to binary traits [12] to traits having ordinal features in an attempt to advance further gene-gene interaction analysis [13].

This meeting, TBC 2012, provided the opportunity for translational bioinformatics researchers to bring together and substantially improve the understanding of complex human diseases for personalizing healthcare. We learned that our 'personal-level' understanding of physiologic and bio-molecular foundations is severely limited. It is clear that many health topics are now within the scope of translational bioinformatics, including complex human disease, medical genetics, cancer, drug repositioning, and response to therapeutic perturbations. There are many barriers to translating our molecular understanding into meaningful products. Effectively integrating biomedical data and knowledge resources with powerful algorithms is even more important than ever. I congratulate the speakers and authors to this conference who are shaping the future of how biomedical informatics translates into better practice. The future of translational bioinformatics, as William Gibson said, is already here, it's just not widely and evenly distributed yet.

## Competing interests

The author declares that they have no competing interests.

## Declarations

TBC 2012 and the publication costs of this research was supported by Basic Science Research Program through the National Research Foundation of Korea (NRF) funded by the Ministry of Education, Science and Technology (2012-0000994).

This article has been published as part of *BMC Medical Genomics *Volume 6 Supplement 2, 2013: Selected articles from the Second Annual Translational Bioinformatics Conference (TBC 2012). The full contents of the supplement are available online at http://www.biomedcentral.com/bmcmedgenomics/supplements/6/S2.
